# Subjective Wellbeing among University Students and Recent Graduates: Evidence from the United Kingdom

**DOI:** 10.3390/ijerph19116911

**Published:** 2022-06-05

**Authors:** William E. Donald, Denise Jackson

**Affiliations:** 1 Southampton Business School, University of Southampton, Southampton SO17 1BJ, UK; 2 Ronin Institute, Montclair, NJ 07043, USA; 3 School of Business and Law, Edith Cowan University, Perth 6027, Australia; d.jackson@ecu.edu.au

**Keywords:** subjective wellbeing, conservation of resources, COVID-19, higher education, policy

## Abstract

This study compares students’ and recent graduates’ perceptions of their subjective wellbeing and offers support mechanisms and resources to enhance wellbeing in higher education. Survey data were collected in September 2021 from 414 UK-based higher education students and recent graduates on their self-perceived subjective wellbeing in March 2020 (before COVID-19 regulations restrictions) and September 2021 (18 months later). Findings showed that subjective wellbeing scores fell for almost three-quarters of university students and recent graduates between March 2020 and September 2021. Interestingly, around one-fifth of participants reported increased subjective wellbeing scores whilst the remaining participants reported no impact. Positive impacts of the pandemic included opportunities for self-improvement with more free time for focusing on health and relationships. Adverse outcomes included feelings of isolation, reduced mental and physical health, difficulties undertaking degree studies and work, travel restrictions, and concerns for labour market competitiveness. The study advances the application of the conservation of resources theory and identifies strategies for higher education institutions to better support and improve their students’ and future graduates’ subjective wellbeing. Strategies include access to counselling, mindfulness, opportunities for participation in hobbies, interaction with peers, flexible work and study options, and guidance on career and finances.

## 1. Introduction

Higher education institutions worldwide face longstanding challenges of mental health issues within their student populations [1]. Elevated mental ill-health following COVID-19 has been reported among university students worldwide, with evidenced rises in the Americas [2], Asia [3], Australia [4], and Europe [5]. This global crisis has been exacerbated by many international students being ‘stranded’ in their study destination country, separated from extended family networks for several months [6]. Consequently, addressing mental health in university populations has been declared a global priority [7] and has led to urgent calls for additional research into the effects of lockdown and broader restrictions on social isolation and student subjective wellbeing [8].

University students have been significantly impacted by the pandemic, with many forced to leave campuses and study online for long periods due to lockdown restrictions [9]. Resultantly, Evans et al. [10] found that around one-third of UK-based students could be classified as clinically depressed, compared to 15% pre-lockdown, and that depression symptoms were highly correlated with worsened sleep quality. Moreover, lockdown restrictions prevented many students and recent graduates from seeking support, with referrals for talking therapies falling by one-third in the first six months of the pandemic due to pressures on the National Health Service [11]. This unprecedented demand has led to concerns of an elevated risk of spiralling mental health issues, as reflected in the aftermath of previous epidemics [12].

Notably, while the pandemic as a crisis can act as a catalyst to address longstanding challenges with student subjective wellbeing [1], policies and ensuant action should be informed by the needs of students and recent graduates to authentically support their subjective wellbeing [4,13,14]. For example, the acquisition of personal resources can be driven by the extent to which students believe themselves to be active and involved citizens within higher education institutions [15]. This study, therefore, seeks to explore and compare the perspectives of 414 university students and recent graduates regarding their subjective wellbeing to inform policy recommendations for higher education institutions. While we analyse subjective wellbeing in the UK context, our findings have global applications for those responsible for managing the mental health of university students.

We posed two research questions. First, how has the COVID-19 pandemic impacted the subjective wellbeing of university students and recent graduates? Second, what support do university students and recent graduates think could be helpful to enhance their subjective wellbeing? The study gauges and compares current students’ and recent graduates’ perspectives on both hedonic and eudaimonic dimensions of their subjective wellbeing before and after the COVID-19 pandemic. It draws on the Conservation of Resources (CoR) theory [16], which can play a vital role in protecting mental health during challenging times, positively framing situations and developing resilience to stress [17]. 

As well as enhancing our theoretical understanding of antecedents of students’ and graduates’ subjective wellbeing and how to support them better, the study’s findings will help inform policy decisions on supporting student mental health and subjective wellbeing within higher education institutions. Exploring and comparing the perceptions of recent graduates and current students will inform policy recommendations that support students during their studies and better prepare them for the transition to work. These policies are essential irrespective of COVID-19, with evidenced growth in mental health issues among university students, recent graduates, and youth more generally before the pandemic [10,18,19]). Further, a reported rise in unhappiness among UK-based children (aged 10 to 15 years) suggests that more subjective wellbeing-related issues could emerge in higher education institutions in the coming years as individuals transition to tertiary education and, subsequently, the graduate labour market [20]. Our focus now moves to review relevant literature on mental health and subjective wellbeing.

## 2. Background Review

### 2.1. Defining and Measuring Subjective Wellbeing

The World Health Organization [21] defines positive mental health as follows:

*A state of well-being in which the individual realizes his or her own abilities, can cope with the normal stresses of life, can work productively and fruitfully, and is able to make a contribution to his or her community.*

Positive mental health is considered synonymous with subjective wellbeing since this acknowledges a shift away from viewing subjective wellbeing through the lens of an absence of mental ill-health [22]. The construct of subjective wellbeing was measured initially via a single item based on the Ladder of Life [23], which is still in use despite concerns about how different people frame their ‘best’ or ‘worst’ possible life [24]. Measures of life satisfaction to gauge subjective wellbeing [25] are no longer used, given the relative insensitivity of ‘satisfaction’ to change [26]. In response, several short scales of subjective wellbeing have emerged, for example, The Warwick-Edinburgh Mental Wellbeing Scale [27] and the Flourishing Scale [28]. However, their non-systematic development and a single summary subjective wellbeing score limit their value in informing policy to target specific subjective wellbeing areas [26,29].

The Ten Features of Positive Wellbeing (TFPW) index comprises a six-item subscale of positive characteristics and a four-item subscale of positive functioning based on a systematic approach to identifying relevant dimensions of subjective wellbeing [30]. Positive characteristics assess hedonic wellbeing—focusing on attaining pleasure and avoiding pain can enhance one’s subjective wellbeing [31]—while positive functioning gauges eudaimonic wellbeing—whereby meaning and authenticity can enhance one’s subjective wellbeing [32]. The TFPW index offers similar overall scores to other short-scale measures of subjective wellbeing, facilitates policy development by identifying specific dimensions that need targeting, and has been validated internationally [29,30,33]. It was, therefore, this study’s chosen measure of subjective wellbeing and is evidenced in Table 1.

### 2.2. Impacts of COVID-19 on Student and Graduate Subjective Wellbeing

COVID-19 has had a significant negative impact on the mental health of many populations worldwide [7], perhaps due to the unpredictability it has caused [3]. University student status was a common risk factor that exacerbated mental distress due to ‘campus closures, cancellation of social events, lower study efficiency with remote online courses, and postponement of exams’ [7], aligning with Australian-based research, which declared universities as high-risk settings for developing mental health issues [18]. Additional studies reported that students were likely to experience feelings of loneliness and isolation from reduced access to the university campus [6], to miss out on opportunities to study abroad that are linked to university completion rates [34], and to experience increased levels of depression [10]. Such views are understandable given that students anticipated they would be socialising and experiencing ‘the best years of their lives’, only for the reality of being isolated in student accommodation and studying remotely with the same tuition fees and living costs as previous cohorts who experienced greater freedoms [6]. 

Whilst the COVID-19 pandemic has increased the risk to mental health in university populations, the challenge pre-dates this global chance event [10]. For example, disruption of academic routines [6] and extensive social media use [35] have been shown to harm students’ mental health. Subjective wellbeing has been associated with perceived employability and psychological capital in India [36] and Turkey [37]. COVID-19 has thus exacerbated pre-existing economic and social disadvantages translating to reduced subjective wellbeing levels [38]. This would likely lead to lower hedonic and eudaimonic wellbeing scores as measured via positive characteristics and positive functioning scores of the TFPW index.

Young people reported lower subjective wellbeing scores worldwide from COVID-19 [39,40], including graduates [41]. Even before the pandemic, graduates already experienced uncertainty and reduced subjective wellbeing from unmet income expectations [42], leading to calls for greater transparency on prospective university students’ employment prospects in declining labour markets [43]. Thus, the pandemic-related restrictions may have also impacted graduates, particularly those working in sectors where remote working was not feasible, such as hospitality and retail [44]. Thus, we propose the following hypotheses:

**Hypothesis** **One** **(H1):**  *Positive characteristics subjective wellbeing scores for university students will be lower since COVID-19 restrictions than before the pandemic.*

**Hypothesis** **Two** **(H2):**  *Positive characteristics subjective wellbeing scores for recent graduates will be lower since COVID-19 restrictions than before the pandemic.*

**Hypothesis** **Three** **(H3):**  *Positive functioning subjective wellbeing scores for university students will be lower since COVID-19 restrictions than before the pandemic.*

**Hypothesis** **Four** **(H4):**  *Positive functioning subjective wellbeing scores for recent graduates will be lower since COVID-19 restrictions than before the pandemic.*

### 2.3. Theoretical Framework

This paper is theoretically framed by the CoR theory, which proposes that increased internal and external resources lead to enhanced subjective wellbeing levels [16]. Here, past experiences and evaluations of the benefits of personal resources and threats of environmental demands can influence subjective wellbeing [45]. Personal resources have been shown to have the most significant potential for determining positive subjective wellbeing outcomes [37], as they can enable individuals to cope with stressful circumstances in their lives [46]. The CoR theory posits that, when our personal resources exceed the resources required to overcome environmental demands, the outcome is reduced stress levels and increased subjective wellbeing [16].

COVID-19 is an example of a global chance event whereby environmental demands place additional pressure on the personal resources of individuals. Moreover, the uncertain nature of the future of work, combined with the risk of further pandemics or alternative global level chance events (e.g., climate change), could lead to environmental demands spiking multiple times during one’s career. The pandemic can thus act as a catalyst to address mental health in students [1] and enable us to understand the impacts of past experiences of university students and recent graduates to inform future policies and strategies [45]. This develops the views of Nimmi et al. [36], who assert that sustainable subjective wellbeing in graduates and across the career span relies on the individual perceiving that they possess the necessary resources to overcome periods of adversity. The position is grounded in positive psychology, whereby positive framing during challenging times—such as COVID-19—can protect one’s mental health [17]. Moreover, positive framing complements the choice of the TFPW index to measure subjective wellbeing by acknowledging that positive mental health sits at the opposite end of a continuum to mental ill-being [30]. The TFPW index uses positive framing of 10 dimensions of criteria used to diagnose anxiety and depression to acknowledge that subjective wellbeing is the opposite of ill-being rather than the absence of ill-being [29].

The CoR lens helps us understand how universities, through student support services (or similar), can support their students in acquiring the necessary resources to navigate challenges and maintain resilience rather than become overwhelmed by environmental demands and the associated threats to their resources.

## 3. Materials and Methods

### 3.1. Participants

In total, 414 UK-based university students and recent graduates of UK universities participated in the study; their characteristics are summarised in Table 2. Students (n = 274) were in their penultimate or final year of undergraduate study, and graduates (n = 178) had completed their undergraduate studies within the previous 12 months, ensuring all participants were in higher education during COVID-19. Gender representation is consistent with national student populations in the UK over the last five years for the overall sample and the student and graduate cohorts [47]. There were no additional restrictions for participation related to university of study, degree course, or current sector of employment. Therefore, data relating to these specific metrics were not collected as part of this study.

### 3.2. Procedures

Participants were invited to complete an online survey in September 2021 to report on their self-perceived subjective wellbeing in early March 2020 (before COVID-19 restrictions) and for September 2021 (18 months since COVID-19 restrictions on daily living in the UK began). Following ethical approval, the survey link was circulated via various social media platforms (e.g., LinkedIn, Facebook, Twitter), online student forums, and online professional networking groups for graduates. The survey link was reposted twice over the four-week data collection period. Participants were asked as part of the informed consent to also confirm their life status as a UK-based student or graduate (with definitions of each provided), and that they had not previously completed the survey.

### 3.3. Measures

The survey instrument included closed- and open-ended questions and comprised three sections, the first capturing student/graduate and gender status. The second section addressed the first research question by collecting data on 10 subjective wellbeing items using the TFPW index [30]. Positive characteristics (six-item scale) measured hedonic aspects of subjective wellbeing: emotional stability, optimism, resilience, positive emotion, self-esteem, and vitality. Positive functioning (four-item scale) measured eudaimonic aspects of subjective wellbeing: competence, engagement, meaning, and positive relationships. 

Participants self-reported a score from 1 to 10 for their perceived subjective wellbeing levels for each item before and since the COVID-19 pandemic restrictions. This is a benefit of the TFPW scale, since scoring out of 10 for 10 items offered percentage subjective wellbeing scores and overcomes the limitations of alternative measures that use Likert scales [22]. 

The final section of the survey presented three open-ended questions. The first and second addressed research question one to gain insights into university students’ and recent graduates’ views of the impact of COVID-19 on their subjective wellbeing: ‘What have been the positive impacts of the COVID-19 pandemic on your subjective wellbeing, if any?’, and ‘What have been the negative impacts of the COVID-19 pandemic on your subjective wellbeing, if any?’ Finally, to address research question two, participants were asked: ‘What support would be helpful to you to enhance your subjective wellbeing following the COVID-19 pandemic, if any?’

### 3.4. Analysis

Rating data were analysed using SPSS 27. Descriptive statistical techniques were used to compute item means and standard deviations before and since the COVID-19 pandemic. Repeated measures ANOVA was used to detect any variations in perceived subjective wellbeing before and since COVID-19 regulations by the student or graduate status. Within- and between-subject main and interaction effects were also examined. 

Responses from the first (n = 349), second (n = 345), and third (n = 318) open-ended questions were analysed in Microsoft Excel. First, we undertook an in-depth review of the written responses to the open questions to ‘engage with those living phenomenon and attempt to understand it from their perspective’ Corley [48]. Next, we developed a codebook by identifying an initial set of codes and then grouping these codes by themes and sub-themes. Finally, we counted the occurrences of university students and recent graduates for each theme and sub-theme to offer summary tables ranked by frequency [49]. This approach to content analysis is termed ‘quantitative dominant’ because it emphasises counting whilst also relying on themes and sub-themes [50]. The hybrid approach recognises that traditional quantitative and qualitative approaches can augment each other and are not mutually exclusive [51].

## 4. Results

### 4.1. Impacts of COVID-19 on Student and Graduate Subjective Wellbeing

Means and standard deviations at item and scale composite levels, before and since the pandemic-related restrictions, are presented for both students and graduates in Table 3. Results indicated that before COVID-19, ‘engagement’, as part of positive functioning, achieved the highest score for both students and graduates. This was followed by ‘optimism’ for students and by ‘positive relationships’ and then ‘optimism’ for graduates. The lowest scores for students before COVID-19 were ‘self-esteem’, ‘vitality’, ‘emotional stability’, and ‘resilience’—all aspects of positive characteristics. The lowest scores for graduates were ‘emotional stability’, ‘self-esteem’, ‘meaning’, and ‘resilience’. Overall, the composite average for positive characteristics was lower for both groups than positive functioning.

While ‘engagement’ still reported the highest mean for students and graduates since the pandemic, ‘optimism’ and ‘positive emotion’ shifted to among the lowest scoring. ‘Emotional stability’ and ‘vitality’ were among the lowest for both groups since the pandemic. The composite mean for positive characteristics remained higher for both groups than ‘positive functioning’, and the gap widened since the pandemic.

To examine H1–H4, a repeated measures ANOVA (α = 0.05) was conducted to test for differences in subjective wellbeing before and after COVID (see Table 4). Cronbach Alpha scores for the TFPW index exceeded 0.80, and Principal Components Analysis indicated that the two scales were unidimensional, with factor loadings ranging from 0.699 to 0.916 for university students and recent graduates. Inter item correlations were calculated for the 10 items, all significant and positive at α = 0.01 level. Correlations ranged from 0.420 to 0.764 before COVID-19, and 0.227 to 0.691 since COVID-19. Normality was examined via skew and kurtosis, with variables falling within the accepted thresholds of 3 and 10, respectively [52].

Within-subject main effects (difference in subjective wellbeing before and since COVID-19 restrictions for all participants) were significant for all items and composite scales other than ‘engagement’. Mean ratings were lower since COVID-19 for all significant items, with effect sizes ranging from small (0.20) to medium (0.50) [53]. The results, therefore, support H1 and H2, given that positive characteristic scores, individually and on average, were significantly lower since COVID-19 restrictions than before the pandemic for both university students and recent graduates. H3 and H4 were partially supported, with three of the four items and the composite average being significantly lower since the pandemic for university students and recent graduates. 

There were no significant ANOVA within-subject interaction effects reported at the item or scale level. This showed that changes to perceptions of subjective wellbeing before and since the pandemic did not differ significantly based on student or graduate status. Between-subject interaction effect results are also reported in Table 4 (variations by the student or graduate status before and since COVID-19). With a Bonferroni correction applied (α = 0.004), there were no significant variations. At the less stringent α = 0.05 level, one can observe significant variations before the pandemic for ‘self-esteem’, ‘vitality’, ‘positive relationships’, ‘positive functioning’, and ‘engagement’ since COVID-19, although effect sizes are small. Mean scores for these subjective wellbeing dimensions were consistently higher for recent graduates than students.

To further explore changes in perceptions from the pandemic, recognising that mean ratings comprise some increases and some decreases, Table 5 presents an analysis of the proportions of students and recent graduates with improved, reduced, and stable scores at the item and scale level. Hedonic subjective wellbeing was perceived to have decreased for around three-in-four university students and recent graduates, whilst eudaimonic subjective wellbeing decreased for just under three-in-five university students and recent graduates. Interestingly, findings showed that 17.52% of university students and 17.14 per cent of recent graduates reported perceived increases in their hedonic subjective wellbeing, whilst 29.20 per cent of university students and 28.57% of recent graduates reported increased eudaimonic subjective wellbeing. The variances supported conducting a content analysis on the positive and negative impacts of the COVID-19 pandemic to compare and contrast university students’ and recent graduates’ perspectives of subjective wellbeing.

The content analysis led to six themes and associated sub-themes for the positive impacts of COVID-19 on subjective wellbeing (Table 6). Counts are included in the table to indicate the number of university students and recent graduates whose responses were aligned with that theme or sub-theme. For brevity, we report some observations and example quotes rather than addressing every theme and sub-theme. The numbers within parentheses relate to the participant that made the quote followed by the sub-theme(s) that it relates to. For example, participant 1 and sub-theme 1a would be presented as (1:1a).

The most significant reported benefit from the pandemic was the opportunity for self-improvement, centring on values, subjective wellbeing, and life focus: ‘I am more open-minded to try out different things such as career change, hobbies, and life goals’ (321:1a); and ‘it has given me the opportunity to grow and reflect on myself as an individual’ (132:1f,1g). This benefit was followed by commentary around gaining more free time, which enabled more time with friends, family, and for self-care: ‘I have had extra time to work on personal development and to spend with my family’ (92:2e,2f); and ‘I have given a lot of time to myself to think, reflect, and visualise what are the things I want’ (375:2c). Some found the pandemic benefitted their study and work arrangements: ‘I was struggling mentally with my work environment and the depression and anxiety that my job was contributing to. Since COVID, I have been able to work from home in an environment I am comfortable in, which has improved my mental health’ (212:3b,5a). Others experienced a strengthening of different forms of relationships: ‘I had the opportunity to spend six months home schooling my four kids during my degree. I worked hard to make it a positive experience for them. Hopefully, they will have good memories—I have’ (31:4c, 4d). Finally, some respondents gained physical health benefits: ‘I have been able to improve habits that lead to positive impacts on my physical health and wellbeing’ (367:5b).

Concerning the most significant differences in percentage points between university students and recent graduates, students reported more time for learning, more family support, and more perceived mental health benefits, whilst graduates reported more time for self-discovery and valuing life by living in the moment.

Our content analysis also produced six themes with sub-themes for the negative impacts of COVID-19 on subjective wellbeing, as seen in Table 7. The most significant negative impact was from isolation and restrictions: ‘I feel that COVID has made me more insecure, isolated, less confident, and more anxious’ (59:7a,7b). This was followed by reduced mental and physical health: ‘I gained weight, my energy went down, I became less outgoing’ (6:8e); and ‘I started to drink and smoke drugs’ (340:8e,8h). There were also concerns of an ‘uncertain future’ (194:8g). Others emphasised challenges with their degree or work, including financial pressures: ‘loss of earnings has a somewhat negative impact on my wellbeing and caused me to stress out more than before the pandemic’ (52:9c).

Regarding the most prominent differences of percentage points between university students and recent graduates, students reported more negative impacts on the labour market, motivation to study, and barriers to studying abroad. In contrast, graduates perceived themselves to be impacted more by job uncertainty, challenges of working from home, delayed start dates for employment, loss of income, and lost opportunities to gain work experience.

### 4.2. Perceived Support Desired to Enhance Subjective Wellbeing

Content analysis also led to seven themes and sub-themes for the perceived support wanted to improve subjective wellbeing (Table 8). Findings show a strong demand for counselling, complementary therapy, and access to mental health support to be provided by universities, employers, and the national health service. Calls for social groups, social activities, and ‘more communities for international students studying abroad’ (399:14b,14f) highlighted the need for more significant interaction following lockdown restrictions. Participants were divided in their preferences for flexible work and study options, with some calling for emphasising ‘a return to campus as soon as possible’ (187:14d), and others expressing a desire ‘for universities to understand that people will be scared to go out and be in classrooms and to consider offering an option of online learning’ (311:16b). Some participants were keen to ‘be free to travel again’ (219:15a), avoid further lockdowns, and see ‘everyone getting the vaccine’ (201:15e). Additionally, in both study and work settings, there were calls for ‘hybrid workdays’ (138:16b), the continuation of ‘flexible working’ (365:16b,16e), and ‘integrated work and study patterns’ (108:16d,16e).

From a career support perspective, students asked for ‘help getting a job’ (3:18a), ‘guidance from mentors’ (122:18a), ‘coaching and self-improvement sessions’ (48:18b), and ‘motivational support’ (387:18b). Participants also called on employers to offer ‘jobs without needing prior experience’ (36:18c) and provide support to ‘reduce the risk of burnout’ (66:19b) via ‘paid stress leave’ (310:19b). Moreover, there was a significant focus on financial support from governments to ‘support students who seem to be the least prioritised population’ (313:19c) and from universities by ‘reducing fees due to not having a traditional university experience’ (20:19d).

Regarding the most considerable differences in percentage points between university students and recent graduates, students favoured university support, job opportunities without the need for prior experience, realistic study workload expectations, mindfulness activities, financial support, and online community group support. In contrast, graduates were more unsure of their preferences but desired opportunities to socialise with friends, improved working conditions such as hybrid work options to minimise commutes, avoidance of delays to start dates for work, improved access to health services, more people opting for vaccine boosters, and additional career mentoring.

Interestingly, additional filtering of the content analysis results showed that proposed support for interventions by students or graduates was not related to whether an individual perceived the COVID-19 pandemic to have had a negative effect, a positive impact, or no effect on their subjective wellbeing.

## 5. Discussion and Implications

Our repeated measures ANOVA findings provide statically significant evidence that the COVID-19 pandemic has negatively impacted subjective wellbeing in most students and graduates, aligning with studies worldwide using various measures of subjective wellbeing [2,4,5]. However, our study provides new insights by capturing positive characteristics (hedonic subjective wellbeing) and positive functioning (eudaimonic subjective wellbeing) before and since the COVID-19 pandemic. Hedonic subjective wellbeing was perceived to have fallen for approximately three-quarters of students and graduates, whilst eudaimonic subjective wellbeing was perceived to have reduced for nearly three-in-five students and graduates. 

Additionally, the repeated measures ANOVA showed some significant differences in scores before and since the COVID-19 pandemic (between-subject effects), but it showed that the pandemic did not impact them directly (within-subject interaction effect). In other words, the pandemic did impact the perceived subjective wellbeing of both students and graduates, but it did not affect students or graduates more strongly than the other. Our findings support observations in the aftermath of previous epidemics exploring mental health impacts on young people [12]. Therefore, universities and organisations can benefit from working collaboratively to achieve win-win outcomes for all stakeholders involved in the university-to-work transition, given the needs of students and graduates [53].

However, almost one-fifth of university students and recent graduates reported perceived increases in their hedonic subjective wellbeing, and just under one-third an increase in eudaimonic subjective wellbeing. The content analysis showed that what was perceived as positive impacts by some participants were perceived as negative impacts by others, highlighting an added level of complexity. Our findings thus support the view from Italy that the impacts of the pandemic are multifaceted, and we respond to calls for additional investigation of these factors in different contexts [54]. 

For example, some participants reported improvements in fitness, health, and sleep, whilst these same aspects worsened for others. Moreover, many of the challenges participants faced were linked to their specific transitionary life stage of study or work. The movement to online classes was beneficial for some students with families and those with long commutes [55], whilst detrimental to others who struggled with the lack of interaction with peers and lecturers [18]. The lack of a private space to study, coupled with loneliness and isolation, reflects previous findings on subjective wellbeing of reduced access to campus [6] and highlights economic and social disadvantages translating to decreasing perceived subjective wellbeing [38]. For recent graduates, the ability to focus on their careers, undertake personal development, and avoid long commutes were positive outcomes for some, whilst for others, this contributed to perceived negative impacts. For example, job uncertainty, delayed start dates for employment, and loss of income were also challenging to some participants, as were long hours and struggles with working from home.

Our findings indicate a series of action points for different university areas regarding the support students and recent graduates perceive could enhance their subjective wellbeing. Providing counselling, mindfulness, and group support are critical for feelings of subjective wellbeing, and student support services (or similar) should strive to ensure that all students can access this support. Student engagement services should also consider enabling students to interact with one another to counter the isolation effects of pandemic-related restrictions [8]. Such opportunities can augment the value of group support by allowing students to share and normalise their experiences. Additionally, given the asserted importance of flexible study arrangements, academics might wish to consider facilitating hybrid approaches to study. These approaches should not hinder students who want to return to campus [9] yet should allow others to continue learning remotely to accommodate specific circumstances, including chronic health conditions or students with long commutes [55]. Providing additional learning support and leeway on assessment submission may also benefit students’ perceived subjective wellbeing.

Our findings suggest that university careers services need to provide more significant support to students and recent graduates. The results align with those of Donald et al. [56], who reported that career counsellors at universities have been experiencing unprecedented demands for services from students and recent graduates since the COVID-19 pandemic. However, the same study found that university careers services often lacked the resources to provide the level of support that students required, suggesting the need for additional investment. Moreover, students desired financial help with their studies via reduced tuition fees or contributions towards living costs since they felt the pandemic had undermined their university experience. This is problematic given the perceived gap between the costs and benefits of higher education in the UK was already narrowing [57].

Employers should also address aspects of recent graduates’ policy recommendations, such as providing hybrid work options and improved working conditions, such as time off, reduced hours, and stress leave. Employers should also consider supporting universities as the benefits of increased subjective wellbeing perceptions can transcend education and workplace boundaries in the same way that negative perceived impacts of the COVID-19 pandemic have impacted both groups [40,41]. Students need to be supported during their studies to identify and acquire specific resources to develop a resource caravan based on their circumstances and experiences [58]. A growth mindset and the habit of accumulating personal resources across the lifespan can enable an individual to develop resilience and navigate difficult circumstances benefiting the individual and their employers [36]. Still, organisations should be providing an environment where individuals can operationalise their resource caravans [36,58], which requires flexibility and investment in physical resources to benefit from improved perceived subjective wellbeing.

The CoR theory is helpful since the pandemic can act as a catalyst to address these ongoing mental health issues prevalent in university students and recent graduates globally [1]. Furthermore, the acquisition of personal resources enables individuals to cope with stressful circumstances in their lives [46] and to manage threats from environmental demands [45], which can determine positive subjective wellbeing outcomes [37]. The ability of university students and recent graduates to be supported to develop personal resources will take on increased significance as the risk of chance events occurring during one’s career span increases [36]. Additionally, encouraging a proactive approach to resource acquisition can help individuals navigate future academic challenges and career shocks by developing resilience and a positive framing mindset rather than succumbing to perceptions of negative subjective wellbeing triggered by environmental constraints.

## 6. Study Limitations and Future Research

Like all research, the study has limitations. It employed a cross-sectional design with self-reported data collected at a single point in time whereby participants self-identified as UK-based students or graduates. Thus, the results are subject to recall bias, relying on participants accurately recalling how they felt before the pandemic in March 2020 and at the point of completing the survey in September 2021. This represents a key limitation since the 18-month recall timespan can impact the ability of an individual to accurately self-report [59]. However, the same timeframe of 18 months was used across all self-reported measures in keeping with recommended guidance [59]. Further, all participants were based in a single country (the UK); data were not restricted to specific universities, degree courses, or sectors of employment; and we only explored one dimension of wellbeing—subjective wellbeing. The sampling approach of distributing the survey link via social media platforms, online student forums, and online professional networking groups for graduates also meant it was not possible to report a response rate for participation as we cannot tell how many of the individuals who viewed the link were illegible to participate in the study. 

Accordingly, future research could adopt a broader perspective and explore other dimensions, such as physical, financial, and spiritual wellbeing. Additional factors could be controlled for, including age, residential status (at home/on campus), and citizenship. Future research may also benefit from comparing individuals from different institutions, degree subjects, or employment sectors. Studies that gather additional demographic data could investigate how wellbeing varies among different groups and geographical locations. For example, there would be value in exploring the views of binary and gender-diverse individuals to inform policy decisions, given that these groups’ adverse mental health outcomes are more pronounced [60]. Finally, a longitudinal study design could explore how university interventions can help students acquire personal resources to support sustainable subjective wellbeing and positive outcomes across their studies and careers.

## 7. Conclusions

This study gives voice to university students and recent graduates by helping higher education institutions make informed policy decisions to better support mental health in their student and graduate populations worldwide [18,19]. Our findings advance the application of positive framing and personal resource development as a strategy to protect one’s mental health [17]. Calls by Burns et al. [61] are also addressed for empirical investigations in a UK context, and the use of the TFPW index offers a preferable measure for policy identification compared with alternative tools [30]. The study further contributes since personal resource acquisition is enhanced when individuals are involved in developing policy approaches [15].

## Figures and Tables

**Table 1:** Ten features of positive wellbeing (TFPW).

Feature	Item
**Positive Characteristics**	
Emotional Stability	I feel relaxed
Optimism	I feel optimistic about the future
Resilience	I deal with problems well
Positive Emotion	I feel cheerful
Self-Esteem	I feel positive about myself
Vitality	I have a lot of energy
**Positive Functioning**	
Competence	I feel a sense of accomplishment
Engagement	I am interested in learning new things
Meaning	I feel my contributions in life are valuable
Positive Relationships	I feel close to other people

The TFPW scale was developed by Huppert et al. [30] and is composed of six items of positive characteristics and four items of positive functioning.

**Table 2:** Participant characteristics (n = 414).

Variable	Group	Count	%
Gender	Female	236	57.00
	Male	178	43.00
Life Status	Student	274	66.18
	Graduate	140	33.82
Sub-groups	Female student	156	37.68
	Male student	118	28.50
	Female graduate	80	19.33
	Male graduate	60	14.49

**Table 3:** Means and standard deviations before and since the COVID-19 pandemic for students and graduates (n = 414).

Before COVID-19					Since COVID-19			
	Students		Graduates		Students		Graduates	
Item	Mean	SD	Mean	SD	Mean	SD	Mean	SD
Emotional Stability	6.68	2.22	7.01	2.21	5.22	2.14	5.29	2.28
Optimism	7.07	2.10	7.33	2.21	5.77	2.11	5.91	2.50
Resilience	6.72	2.08	7.11	1.99	6.06	2.18	6.32	2.21
Positive Emotion	6.99	2.16	7.17	2.13	5.81	2.19	5.87	2.16
Self-Esteem	6.64	2.17	7.09	2.35	6.09	2.33	6.29	2.37
Vitality	6.67	2.27	7.25	2.10	5.49	2.24	5.69	2.44
Competence	6.73	2.05	7.13	2.09	6.26	1.99	6.48	2.12
Engagement	7.24	1.90	7.72	1.94	7.08	2.04	7.59	1.91
Meaning	6.73	2.05	7.09	2.16	6.33	2.12	6.44	2.19
Positive Relationships	6.82	2.22	7.36	2.23	5.67	2.55	6.04	2.44
Positive Characteristics (composite)	6.79	1.84	7.16	1.89	5.74	1.76	5.89	1.89
Positive Functioning (composite)	6.88	1.71	7.33	1.79	6.34	1.77	6.64	1.65

Positive Characteristics captures six items (Emotional Stability, Optimism, Resilience, Positive Emotion, Self-Esteem, and Vitality). Positive Functioning captures four items (Competence, Engagement, Meaning, and Positive Relationships).

**Table 4:** ANOVA within- and between-subject main and interaction effects for COVID and student/graduate status.

	Within-Subject Main Effect (COVID)				Within-Subject Interaction Effect (COVID & STATUS)				Between-Subject Interaction Effect (STATUS & Before COVID)				Between-Subject Interaction Effect (STATUS & Since COVID)			
Variable	df	F	*p*	η2	df	F	*p*	η2	df	F	*p*	η2	df	F	*p*	η2
Emotional Stability	412	152.664	<0.001	0.270	412	0.971	0.325	0.002	412	1.990	0.159	0.005	412	0.096	0.757	0.000
Optimism	412	115.349	<0.001	0.219	412	0.247	0.619	0.001	412	1.403	0.237	0.003	412	0.344	0.558	0.001
Resilience	412	49.325	<0.001	0.107	412	0.434	0.511	0.001	412	3.449	0.064	0.008	412	1.302	0.254	0.003
Positive Emotion	412	106.923	<0.001	0.206	412	0.240	0.625	0.001	412	.639	0.424	0.002	412	0.073	0.787	0.000
Self-Esteem	412	36.527	<0.001	0.081	412	1.343	0.247	0.003	412	3.828	0.051	0.009	412	0.638	0.425	0.002
Vitality	412	108.539	<0.001	0.209	412	2.075	0.150	0.005	412	6.407	0.012	0.015	412	0.721	0.396	0.002
Competence	412	21.480	<0.001	0.050	412	0.575	0.449	0.001	412	3.506	0.062	0.008	412	1.079	0.299	0.003
Engagement	412	1.676	0.196	0.004	412	0.009	0.925	0.000	412	5.861	0.016	0.014	412	5.864	0.016	0.014
Meaning	412	22.194	<0.001	0.051	412	1.332	0.249	0.003	412	2.862	0.091	0.007	412	0.248	0.619	0.001
Positive Relationships	412	67.411	<0.001	0.141	412	0.311	0.577	0.001	412	5.462	0.020	0.013	412	2.023	0.156	0.005
Positive Characteristics (composite)	412	155.902	<0.001	0.275	412	1.299	0.255	0.003	412	3.621	0.058	0.009	412	0.680	0.410	0.002
Positive Functioning (composite)	412	42.078	<0.001	0.093	412	.594	0.441	0.001	412	6.136	0.014	0.015	412	2.796	0.095	0.007

**Table 5:** Proportions of score changes before and since COVID-19 at item and scale level (n = 414).

	University Student (n = 274)						Graduate (n = 140)					
	Decrease		Same		Increase		Decrease		Same		Increase	
Variable	n	%	n	%	n	%	n	%	n	%	n	%
Emotional Stability	189	68.98	45	16.42	40	14.60	97	69.29	24	17.14	19	13.57
Optimism	168	61.31	66	24.09	40	14.60	85	60.71	32	22.86	23	16.43
Resilience	115	41.97	116	42.34	43	15.69	68	48.57	49	35.00	23	16.43
Positive Emotion	165	60.22	72	26.28	37	13.50	83	59.29	41	29.29	16	11.43
Self-Esteem	121	44.16	91	33.21	62	22.63	72	51.43	48	34.29	20	14.29
Vitality	153	55.84	86	31.39	35	12.77	87	62.14	39	27.86	14	10.00
Competence	127	46.35	83	30.29	64	23.36	67	47.86	34	24.29	39	27.86
Engagement	95	34.67	108	39.42	71	25.91	43	30.71	61	43.57	36	25.71
Meaning	106	38.69	113	41.24	55	20.07	61	43.57	58	41.43	21	15.00
Positive Relationships	146	53.28	68	24.82	60	21.90	81	57.86	29	20.71	30	21.43
Positive Characteristics (composite)	208	75.91	18	6.57	48	17.52	102	72.86	14	10.00	24	17.14
Positive Functioning (composite)	162	59.12	32	11.68	80	29.20	81	57.86	19	13.57	40	28.57
Overall	201	73.36	12	4.38	61	22.26	100	71.43	11	7.86	29	20.71

**Table 6:** Perceived positive outcomes of COVID-19 on subjective wellbeing (n = 349).

Themes and Sub-Themes	Total		Students		Graduates	
	n	%	n	%	n	%
**Theme 1: Opportunities for self-improvement**	**144**		**88**		**56**	
1a. Growth/development: knowledge/hobbies	33	22.92	22	25.00	11	19.65
1b. Improved health/wellbeing	32	22.22	20	22.73	12	21.43
1c. Improved self-awareness/quality of life	28	19.44	19	21.59	9	16.07
1d. Value life: live in the moment/revaluate	19	13.19	8	9.09	11	19.64
1e. More focused: proactive/motivated/determined	11	7.64	8	9.09	3	5.36
1f. Reflect on what is important	11	7.64	7	7.95	4	7.14
1g. Focus on self/faith/what I want	10	6.95	4	4.55	6	10.71
**Theme 2: More free time**	**112**		**78**		**34**	
2a. Time with friends/family/partners	30	26.79	22	28.21	8	23.53
2b. Time for self-care: relax/rest/slow down	26	23.21	18	23.08	8	23.53
2c. Time for self-discovery: reflect/plan/visualise	20	17.86	10	12.82	10	29.41
2d. Time for learning: skills/leisure/hobbies	19	16.96	17	21.79	2	5.88
2e. Time at home: free time/break/alone time	11	9.82	9	11.54	2	5.88
2f. Time for self-improvement: habits/discipline	6	5.36	2	2.56	4	11.76
**Theme 3: Studies and work**	**96**		**64**		**32**	
3a. Learning: studies/skills/convenience/support	46	47.92	29	45.31	17	53.13
3b. Conditions: no commute/flexibility/productive	36	37.50	25	39.06	11	34.38
3c. Finances: fewer expenses/saving money	11	11.45	9	14.06	2	6.25
3d. Career focus: revaluate career/career-driven	3	3.13	1	1.56	2	6.25
**Theme 4: Relationships**	**81**		**58**		**23**	
4a. Family: family support/communication	51	62.97	39	67.24	12	52.17
4b. Friendships: closer to people/friends online	16	19.75	10	17.24	6	26.09
4c. More time at home	8	9.87	5	8.62	3	13.04
4d. Improved relationships	4	4.94	3	5.17	1	4.35
4e. More time with pets	2	2.47	1	1.72	1	4.35
**Theme 5: Fitness and health**	**77**		**53**		**24**	
5a. Mental health: energy/resilience/relaxed	42	54.54	31	58.49	11	45.83
5b. Physical health/fitness	21	27.27	13	24.53	8	33.33
5c. Sleep/more rest	9	11.69	6	11.32	3	12.50
5d. Hygiene	3	3.90	1	1.89	2	8.33
5e. Not catching COVID-19	2	2.60	2	3.77	0	0.00
**Theme 6: None**	**22**		**17**		**5**	
6a. No positive impacts	22	100	17	100	5	100

**Table 7:** Perceived negative impacts of COVID-19 on subjective wellbeing (n = 345).

Themes and Sub-Themes	Total		Students		Graduates	
	n	%	n	%	n	%
**Theme 7: Isolation and restrictions**	**198**		**135**		**63**	
7a. Isolation and reduced/no social interaction	127	64.13	87	64.44	40	63.49
7b. Decrease in social skills/relationship struggles	31	15.66	23	17.04	8	12.70
7c. Travel restrictions	20	10.10	14	10.37	6	9.52
7d. Reduced quality of life	9	4.55	4	2.96	5	7.94
7e. Distrust of government/media	7	3.54	4	2.96	3	4.76
7f. Division: differing views of COVID-19	4	2.02	3	2.22	1	1.59
**Theme 8: Reduced mental and physical health**	**188**		**131**		**57**	
8a. Mental health issues	60	31.91	41	31.30	19	33.33
8b. Loss of control: insecure/less confident	44	23.41	32	24.43	12	21.05
8c. Less active: gym closed/poorer physical health	18	9.57	13	9.92	5	8.77
8d. Suffering: abuse at home/death of loved ones	18	9.57	9	6.87	9	15.79
8e. Bad habits: smoking/drinking/drugs/weight	14	7.45	9	6.87	5	8.77
8f. Lack of motivation	13	6.91	11	8.40	2	3.51
8g. Uncertainty: feeling unsafe/worried	9	4.79	7	5.34	2	3.51
8h. Negative impact on health/wellbeing	8	4.26	5	3.82	3	5.26
8i. Physical impact: pain/wearing masks	4	2.13	4	3.05	0	0.00
**Theme 9: Degree and work**	**55**		**39**		**16**	
9a. Study: less motivated/lower grades/harder	22	40.00	20	51.28	2	12.50
9b. Loss of work experience	13	23.64	8	20.51	5	31.25
9c. Loss of income/financial problems	11	20.00	6	15.38	5	31.25
9d. Work from home: low efficiency/more hours	6	10.91	2	5.13	4	25.00
9e. Online environment: technology issues	3	5.45	3	7.69	0	0.00
**Theme 10: Travel**	**26**		**17**		**9**	
10a. Feeling distant from others: less connected	10	38.46	6	35.29	4	44.44
10b. Travel interference/restrictions	10	38.46	7	41.18	3	33.33
10c. Reduced freedom of movement	4	15.39	2	11.76	2	22.22
10d. Study abroad plans changed	2	7.69	2	11.76	0	0.00
**Theme 11: Labour market**	**18**		**7**		**11**	
11a. Poor local labour market	7	38.89	5	71.43	2	18.18
11b. Job loss/reduced hours	6	33.33	2	28.57	4	36.36
11c. Job uncertainty: cancelled career programmes	3	16.67	0	0.00	3	27.27
11d. Job delays: delayed selection processes/start date	2	11.11	0	0.00	2	18.18
**Theme 12: None**	**11**		**7**		**4**	
12a. No negative impacts	11	100	7	100	4	100

**Table 8:** Perceived support required to enhance subjective wellbeing (n = 318).

Themes and Sub-Themes	Total		Students		Graduates	
	n	%	n	%	n	%
**Theme 13: Counselling, mindfulness, and support**	**99**		**74**		**25**	
13a. Counselling: mental health support	57	57.58	44	59.46	13	52.00
13b. Self-care strategies: sleep/planning/groups	13	13.13	9	12.16	4	16.00
13c. General support: university/employer	12	12.12	10	13.51	2	8.00
13d. Mindfulness: techniques/strategies/meditation	9	9.09	9	12.16	0	0.00
13e. Health services: improved information/access	7	7.07	2	2.70	5	20.00
13f. Talking to close friends/family	1	1.01	0	0.00	1	4.00
**Theme 14: Opportunities for hobbies and interactions**	**96**		**65**		**31**	
14a. Interactions: socialise/connect with friends	30	31.25	16	24.62	14	45.16
14b. Interactions: online groups/community	29	30.21	22	33.85	7	22.58
14c. Increase activities: holidays/travel/exercise	24	25.00	17	26.15	7	22.58
14d. Return to normal/opening of facilities	6	6.25	5	7.69	1	3.23
14e. Entertainment: videos/social activities	5	5.21	4	6.15	1	3.23
14f. Cooperation: peace/harmony/respect/spiritual	2	2.08	1	1.54	1	3.23
**Theme 15: Vaccine, testing, and guidelines**	**56**		**34**		**22**	
15a. Easing restrictions: able to travel	18	32.15	12	35.29	6	27.27
15b. Guidelines: country level and better controlled	13	23.21	8	23.53	5	22.73
15c. Transparent government/media messaging	10	17.86	6	17.65	4	18.18
15d. Support from government and business	6	10.71	4	11.76	2	9.09
15e. Vaccine/booster uptake and more testing	7	12.50	2	5.88	5	22.73
15f. Other approaches	2	3.57	2	5.88	0	0.00
**Theme 16: Flexible options for work and study**	**50**		**39**		**11**	
16a. University support	18	36.00	17	43.59	1	9.09
16b. Hybrid work and study options	12	24.00	9	23.08	3	27.27
16c. Unsure of preference	8	16.00	4	10.26	4	36.36
16d. Realistic study workload expectations	6	12.00	6	15.38	0	0.00
16e. Working conditions: commuting/time off	6	12.00	3	7.69	3	27.27
**Theme 17: None**	**50**		**33**		**17**	
17a. Not sure/not needed	50	100	33	100	17	100
**Theme 18: Career guidance**	**31**		**18**		**13**	
18a. Career support and mentoring	14	45.16	7	38.89	7	53.85
18b. Support groups	14	45.16	8	44.44	6	46.15
18c. Job opportunities without the need for experience	3	9.68	3	16.67	0	0.00
11d. Job delays: delayed selection processes/start date	2	11.11	0	0.00	2	18.18
**Theme 19: Financial support**	**20**		**11**		**9**	
19a. Financial support	8	40.00	5	45.45	3	33.33
19b. Employment: reduced hours/stress leave/bonus	6	30.00	3	27.27	3	33.33
19c. Government support: financial/food/health	4	20.00	2	18.18	2	22.22
19d. Reduced university fees (experience not same)	2	10.00	1	9.09	1	11.11

## Data Availability

Access to the dataset is not possible due to ethical approval restrictions.
